# Unveiling biomarkers of telitacicept’s efficacy in SLE treatment through proteomics and metabolomics

**DOI:** 10.3389/fimmu.2026.1779880

**Published:** 2026-03-04

**Authors:** Huiyu Nie, Siyuan Chang, Hanhan Chen, Jiahui Shi, Shu Li, Xiaofei Peng, Wei Cheng, Jia Wang, Qi Tang, Yan Ge, Xi Xie, Fen Li

**Affiliations:** 1 Department of Rheumatology and Immunology, The Second Xiangya Hospital of Central South University, Changsha, Hunan, China; 2 Clinical Medical Research Center for Systemic Autoimmune Diseases, Changsha, Hunan, China

**Keywords:** biomarker, metabolomics, proteomics, systemic lupus erythematosus, telitacicept

## Abstract

**Background:**

The pathogenesis of systemic lupus erythematosus (SLE) is closely associated with abnormal activation of B lymphocytes. Telitacicept simultaneously blocks B-cell stimulating factors and proliferation-inducing ligands, thereby inhibiting B-cell proliferation and differentiation, demonstrating favorable therapeutic efficacy in the majority of SLE patients. However, there is a lack of reliable biomarkers of efficacy and systematic elucidation of its mechanism of action.

**Methods:**

The study employed proteomics and metabolomics analysis to explore biomarkers and mechanisms underlying therapeutic response variability to Telitacicept in SLE patients. Twenty-five SLE patients were enrolled and divided into the responder group and non-responder group based on the SLE Response Index 4 to identify key proteins, metabolites, and mechanisms associated with treatment response.

**Results:**

Proteomics results revealed XPNPEP3, SRSF5, SRSF6, WARS1, IDH1, and ITLN1 as protein biomarkers correlated with Telitacicept efficacy in SLE patients. Metabolomics results indicated that pyruvate was a potential metabolic biomarker for responder group, while gamma-aminobutyric acid (GABA) was a potential biomarker for non-responder group. The combined analysis revealed that both pyruvate and IDH1 participate in the citric acid cycle. GABA showed a negative correlation with XPNPEP3.

**Conclusions:**

The above results reveal biomarkers related to the differential efficacy of Telitacicept in treating SLE patients and potential mechanisms underlying these differences, which may provide a reference for personalized treatment and mechanistic research in SLE.

## 1 Introduction

Systemic lupus erythematosus (SLE) is an autoimmune disease characterized by heterogeneous clinical presentations and significant therapeutic challenges, with its hallmark being the butterfly rash frequently accompanied by fatigue, fever, joint pain, swelling, morning stiffness, proteinuria, and hematuria (1). Globally, the disease affects approximately 43.7 per 100,000 individuals (2). Advances in medical technology and increased disease awareness have contributed to a progressive rise in the diagnosis rate, enabling timely interventions. Nevertheless, due to the complex etiology of SLE and significant individual variations, optimizing treatment regimens and improving patient quality of life remain pressing concerns in the medical community.

It has been revealed that SLE patients have a large number of abnormally activated B cells capable of producing multiple autoantibodies, such as antinuclear antibodies (ANA) and anti-double-stranded DNA (dsDNA) antibodies, playing a crucial role in the onset and progression of the disease (3). Advances in immunology and molecular biology have elucidated the mechanisms underlying abnormal B cell activation, including elevated levels of B cell-activating factor (BAFF), enhanced B cell receptor signaling, and dysregulation of immunomodulatory functions (4, 5). Additionally, a proliferating inducing ligand (APRIL), a pivotal cytokine in B cell activation, interacts with the B cell surface transmembrane protein activator and the calmodulin-cyclooxygenase ligand-binding molecule to participate in T cell-independent antibody responses and B cell regulatory class switching, and binds to the B cell maturation antigen on the surface of plasma cells, maintaining their steady-state survival (6). These insights underscore the therapeutic potential of B cell-targeted strategies, which alleviate disease activity and reduce organ damage by suppressing abnormal B-cell activation and decreasing the production of autoantibodies.

Recent advances in B-cell-targeted biologics have transformed SLE management, with agents such as Belimumab (7), Rituximab (8), and Telitacicept (9). Notably, Telitacicept, an innovative drug independently developed in China, is a dual-targeted B-cell biologic that simultaneously acts on both BAFF and APRIL targets and has received conditional marketing approval in China based on compelling clinical trial results and therapeutic potential (10). An observational study demonstrated the favorable therapeutic effects of Telitacicept on SLE patients, effectively reducing levels of IgM and 24-hour urinary protein (11). A single-center, retrospective, real-world study indicated that Telitacicept could reduce the Systemic Lupus Erythematosus Disease Activity Index (SLEDAI), Physician Global Assessment (PGA) scores, and British Isles Lupus Assessment Group (BILAG) indices, while increasing the SLE responder index 4 (SRI-4) response rate (12).

**Figure 1:**
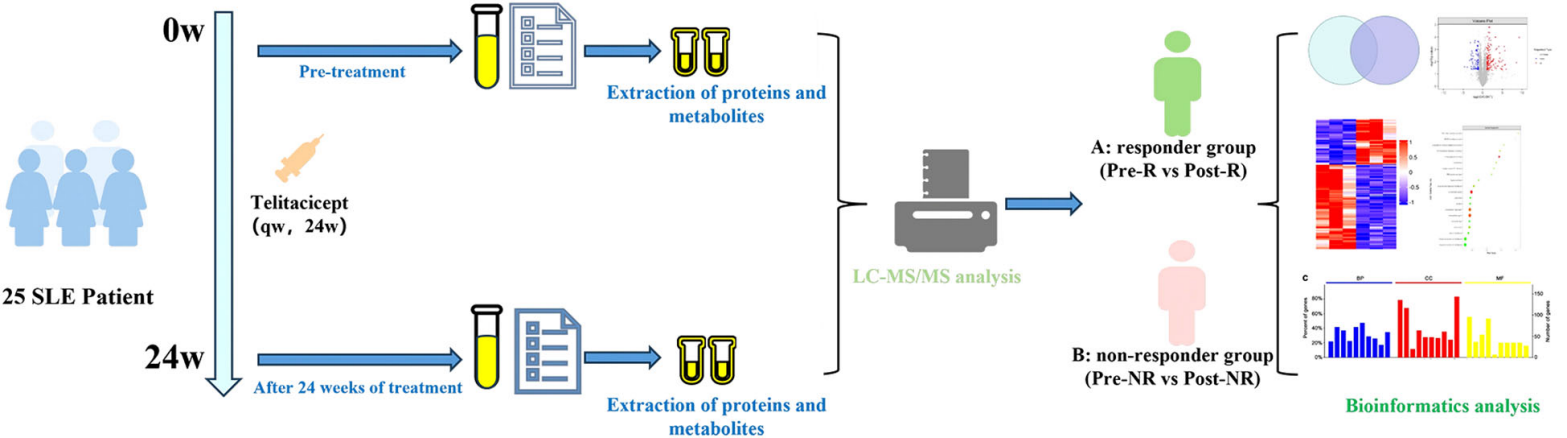
The workflow of the study.

Currently, numerous studies have employed omics technologies to explore SLE in depth (13), but in-depth analysis of drug treatment efficacy remains relatively lacking. In this study, we employed Astral-DIA proteomics and UHPLC non-targeted metabolomics technologies to investigate the changes in serum proteins and metabolites of SLE patients treated with Telitacicept, aiming to identify therapeutic efficacy-related protein and metabolite biomarkers and explore the mechanisms underlying treatment response variability (**Figure 1**).

## 2 Methods

### 2.1 Study participants

This study included 25 patients with SLE treated with Telitacicept at the Second Xiangya Hospital of Central South University, from December 31, 2022, to May 31, 2024, based on the 1997 American College of Rheumatology (ACR) classification criteria (14). The specified inclusion and exclusion criteria were list in **Supplementary Table 1**. This study was conducted in accordance with the Declaration of Helsinki (15) and was approved by the Clinical Research Ethics Committee of Second Xiangya Hospital of Central South University (Ethics Approval Number: LYF2022151). All SLE patients signed informed consent forms prior to treatment.

### 2.2 Treatment regimen and efficacy evaluation

This study references the Phase III clinical trial protocol for Telitacicept in China (NCT06456567). Patients received standard therapy plus regular administration of 80 mg Telitacicept subcutaneous injection once week for 24 weeks. Clinical data collection occurred at Week 0 (baseline) and at the end of Week 24 of treatment, and serum samples were also collected for proteomics and metabolomics sequencing. The standard treatment regimen involves the stable use of any two of the following: glucocorticoids (methylprednisolone or prednisone), antimalarials (hydroxychloroquine), and immunosuppressants (including mycophenolate mofetil, tacrolimus, cyclosporine, azathioprine, cyclophosphamide, methotrexate, leflunomide, etc.).

According to the primary efficacy endpoint in the Phase III clinical trial protocol for Telitacicept, this study adopted SRI-4 as the efficacy assessment standard (16). SRI-4 comprises three core components: the SLEDAI score (17), BILAG score (18), and PGA score (19). SRI-4 response criteria are defined as follows: (1) A decrease in SLEDAI score of ≥4 points; (2) No new organs reaching grade A on the BILAG score, or fewer than 2 new organs reaching grade B; (3) An increase in PGA score of no more than 0.3 points compared to baseline. According to the SRI-4 response criteria, SLE patients fulfilling the criteria are categorized as the responder (R) group, while those failing to meet the criteria are classified as the non-responder (NR) group. For pre-post treatment comparisons, subgroups are defined as follows: R group includes Pre-treatment responder (Pre-R) group and Post-treatment responder (Post-R) group; NR group comprises Pre-treatment non-responder (Pre-NR) group and Post-treatment non-responder (Post-NR) group.

### 2.3 Clinical data statistics

Clinical data of SLE patients were collected from the outpatient system of Second Xiangya Hospital of Central South University. Statistical analysis was performed using SPSS software (IBM SPSS Statistics for Windows, Version 26.0). The pairwise t-test was used for normally distributed variables; otherwise, the Wilcoxon signed-rank test was applied. For all quantitative data, descriptive statistics were presented as mean ± standard deviation (X ± SD) for normally distribution, or as median and interquartile range [M (P25, P75)] for skewed distribution.

### 2.4 Proteomics analysis

The proteomics analysis primarily includes protein extraction, peptide digestion, liquid chromatography-tandem mass spectrometry (LC-MS/MS) data acquisition, and database searching (20). The peptides were analyzed by LC-MS/MS in data independent acquisition (DIA) mode. The DIA data were processed using DIA-NN software with the following parameter settings: enzyme of trypsin, max miss cleavage site of 1, fixed modification of Carbamidomethyl(C), and the dynamic modifications of Oxidation(M) and Acetyl(Protein N-term). Subsequently, the mass spectrometry results underwent bioinformatics analysis, primarily comprising differential expression analysis and functional analysis. The pairwise t-test was used to compare the differences in protein abundance before and after treatment (FC >1.5 or <0.67, and P value <0.05) (21, 22). Benjamini and Hochberg procedure was applied to adjust for multiple hypothesis testing. The functional analysis included subcellular localization analysis, domain analysis, and transcription factor analysis. Finally, key differentially expressed proteins associated with therapeutic efficacy were screened, followed by receiver operating characteristic (ROC) curve analysis, area under the curve (AUC) calculation, and correlation analysis, exploring the association between the expression of these key proteins and clinical indicators, as well as their biological significance.

### 2.5 Metabolomics analysis

The metabolomics analysis primarily involves metabolite extraction and chromatography-mass spectrometry analysis. Serum samples were subjected to chromatographic analysis using Vanquish LC ultra-high performance liquid chromatography (UHPLC) system, followed by mass spectrometry using an Orbitrap Exploris™ 480 mass spectrometer. Detection was performed in both positive ion mode (POS) and negative ion mode (NEG) using electrospray ionization. After the raw data were converted to mzXML format by ProteoWizard, the XCMS package was used for peak alignment, retention time correction, and peak area extraction, followed by metabolites identification, data preprocessing, and data quality evaluation. Subsequently, the mass spectrometry results underwent bioinformatics analysis, primarily comprising differential expression analysis and pathway enrichment analysis. The significantly differential metabolites were determined based on the VIP obtained by the orthogonal partial least squares discriminate analysis (OPLS-DA) model and the P value of pairwise t test. Benjamini and Hochberg procedure was applied to adjust for multiple hypothesis testing. VIP >1 and P value <0.05 were considered significantly differential metabolites (23, 24). The pathway enrichment analysis included Kyoto Encyclopedia of Genes and Genomes (KEGG) enrichment analysis (25) and metabolite set enrichment analysis (MSEA). KEGG enrichment analysis is used to explore the primary metabolic pathways within the target metabolic set, but it has certain limitations. MSEA can compensate for these shortcomings by identifying metabolites and their pathways that exhibit low abundance changes yet remain significant for disease regulation. Finally, key differentially expressed metabolites suggesting therapeutic efficacy are screened, followed by ROC analysis and correlation analysis to investigate the association between the expression of these key metabolites and clinical indicators, as well as their biological significance.

### 2.6 Integrated analysis of proteomics and metabolomics

KEGG pathway analysis and correlation analysis were performed on differentially expressed proteins and metabolites. Pathway analysis, based on KEGG annotation, utilized venn diagrams to illustrate pathways jointly involved in both proteins and metabolites. Correlation analysis involved log_2_ normalization of quantitative data from differentially expressed proteins and metabolites and construction of pearson correlation-based hierarchical clustering heatmaps using the pheatmap R package (Version 1.0.12).

## 3 Results

### 3.1 Demographic and clinical characteristics

After 24 weeks of treatment, 20 patients achieved an SRI-4 response according to the SRI-4 criteria, while 5 patients did not meet the criteria, resulting in an SRI-4 response rate of 80%. Patient demographics, including organ involvement and laboratory test results, were summarized in **Table 1**. Comparisons between pre- and post-treatment in the R group of SLE patients revealed statistically significant changes in several clinical indicators, including SLEDAI scores, PGA scores, anti-dsDNA antibodies, albumin, globulin, urinary albumin, total urinary protein, urinary albumin/creatinine ratio, total urinary protein/creatinine ratio, erythrocyte sedimentation rate (ESR), C-reactive protein (CRP), C3, and IgG. In the comparison of the NR group, only the change in CRP levels showed statistical significance in the clinical indicators. However, the CRP levels increased after treatment, consistent with the outcome of treatment failure. These findings indicate that Telitacicept can effectively improve clinical symptoms and laboratory data in the majority of SLE patients.

**Table 1:** Clinical information of SLE patients before and after Telitacicept treatment.

Parameters	Responder group (n=20)		Non-responder group (n=5)		*P*	
	Baseline	24 weeks	Baseline	24 weeks	*P* ^R^	*P* ^NR^
Basic Information						
Age (years)	37 ± 10	38 ± 10	31 ± 12	32 ± 12	>0.999	>0.999
Female/Male (n)	20/0	20/0	5/0	5/0	>0.999	>0.999
Duration (months)	122 ± 69	128 ± 69	95 ± 60	101 ± 60	>0.999	>0.999
SLEDAI (score)	13 ± 6	7 ± 6	15 (10, 19)	14 (9, 19)	**<0.001**	0.214
PGA (score)	2.2 ± 0.5	1.5 ± 0.4	2.5 (1.5, 2.9)	2.2 (1.5, 2.8)	**<0.001**	0.221
BILAG organ involvement, n (%)	20 (100)	5 (25)	5 (100)	5 (100)	**0.021**	>0.999
System involvement						
Skin, n (%)	8 (40)	1 (5)	1 (20)	1 (20)	**0.008**	>0.999
Serositis, n (%)	2 (10)	1 (5)	1 (20)	1 (20)	0.317	>0.999
Kidney, n (%)	14 (70)	14 (70)	3 (60)	3 (60)	>0.999	>0.999
Nervous system, n (%)	2 (10)	2 (10)	3 (60)	3 (60)	>0.999	>0.999
Blood system, n (%)	4 (20)	2 (10)	0 (0)	0 (0)	0.157	>0.999
Clinical index						
ANA (+), n (%)	20 (100)	19 (95)	5 (100)	5 (100)	0.317	>0.999
Anti-dsDNA antibody (+), n (%)	17 (85)	10 (50)	3 (60)	3 (60)	**0.008**	>0.999
Anti-Sm antibody (+), n (%)	9 (45)	7 (35)	2 (40)	2 (40)	0.157	>0.999
ALB (g/L)	34.8 ± 6.2	41.2 ± 4.3	37.1 ± 11.5	36.7 ± 8.8	**0.001**	0.886
GLO (g/L)	26.2 ± 5.1	23.5 ± 3.5	32.1 ± 14.2	31.5 ± 13.9	**0.016**	0.782
UALB (mg/L)	2577.0 ± 2202.9	689.4 ± 1018.9	3011.6 ± 2423.8	2943.8 ± 3977.7	**0.010**	0.977
UTP (mg/L)	3580.8 ± 3134.2	1048.1 ± 1470.8	3641.3 ± 2899.2	3633.0 ± 4829.6	**0.013**	0.998
UACR (mg/g)	1619.3 ± 1275.2	358.3 ± 463.7	2291.0 ± 1779.9	2733.0 ± 2547.7	**0.004**	0.643
UTPCR (mg/g)	2207.2 ± 1615.8	543.7 ± 644.0	2771.3 ± 2110.1	3308.6 ± 3109.8	**0.002**	0.672
ESR (mm/h)	29 ± 20	13 ± 8	35 ± 28	35 ± 34	**0.004**	0.947
CRP (mg/L)	4.3 ± 3.8	2.1 ± 2.2	1.2 ± 0.8	3.0 ± 2.0	**0.045**	0.044
C3 (g/L)	0.7 ± 0.3	0.9 ± 0.2	0.8 ± 0.4	0.7 ± 0.4	**0.001**	0.349
IgG (g/L)	12.6 (10.6, 16.4)	9.3 (6.9, 10.6)	23.7 ± 21.5	22.2 ± 19.5	**<0.001**	0.326
Clinical treatment						
Telitacicept, n (%)	20 (100)	20 (100)	5 (100)	5 (100)	>0.999	>0.999
GC, n (%)	20 (100)	20 (100)	5 (100)	5 (100)	>0.999	>0.999
HCQ, n (%)	19 (95)	19 (95)	4 (80)	4 (80)	>0.999	>0.999
MMF, n (%)	15 (75)	15 (75)	4 (80)	4 (80)	>0.999	>0.999
Tac, n (%)	1 (5)	1 (5)	3 (60)	3 (60)	>0.999	>0.999

SLE, systemic lupus erythematosus; SLEDAI, Systemic Lupus Erythematosus Disease Activity Index; PGA, Physician Global Assessment; BILAG, British Isles Lupus Assessment Group; ANA, antinuclear antibody; ALB, albumin; GLO, globulin; UALB, urinary albumin; UTP, urinary total protein; UACR, urinary albumin/creatinine ratio; UTPCR, urinary total protein/creatinine ratio; ESR, erythrocyte sedimentation rate; CRP, C-reactive protein; GC, glucocorticoid; HCQ, hydroxychloroquine; MMF, mycophenolate mofetil; Tac, Tacrolimus; *P*^R^, Comparison of the responder group baseline and treatment at 24 weeks; *P*^NR^, Comparison of the non-responder group baseline and treatment at 24 weeks. Bolded numbers indicate values with P < 0.05.

### 3.2 Effect of telitacicept treatment on serum proteins in SLE patients

**Figure 2:**
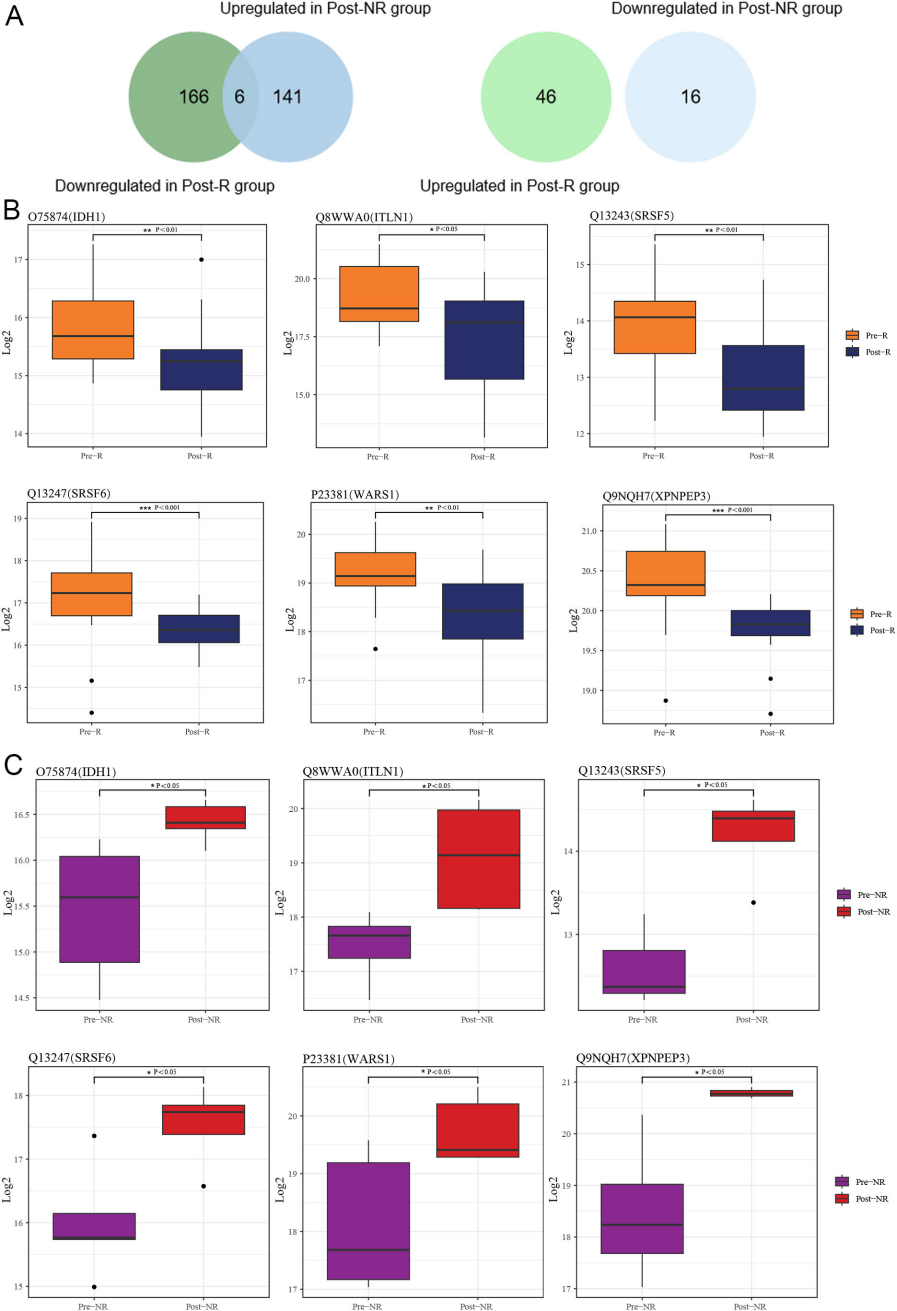
Identification of key differentially expressed proteins in SLE patients treatment with Telitacicept. **(A)** The venn diagrams showed the common proteins between the Pre-R vs Post-R and Pre-NR vs Post-NR groups. **(B)** The box plot showed the expression levels of six key proteins before and after treatment in the R group. **(C)** The box plot showed the expression levels of six key proteins before and after treatment in the NR group. * : P<0.05, ** : P<0.01, *** : P<0.001.

With FC > 1.5 or < 0.67 and P value < 0.05, 218 (46 upregulated and 172 downregulated) and 163 (147 upregulated and 16 downregulated) differentially expressed proteins were identified in the Pre-R vs Post-R group and Pre-NR vs Post-NR group, respectively (**Supplementary Table 2**). To identify key proteins indicative of therapeutic efficacy, 172 proteins lowly expressed in Post-R were intersected with 147 proteins highly expressed in Post-NR, and 46 proteins highly expressed in Post-R were intersected with 16 proteins lowly expressed in Post-NR (**Figure 2A**). Results indicate that only six common proteins were identified: Q9NQH7 (XPNPEP3), Q13243 (SRSF5), Q13247 (SRSF6), P23381 (WARS1), O75874 (IDH1), and Q8WWA0 (ITLN1). As shown in **Figures 2B, C**, these six proteins were downregulated in the R group and upregulated in the NR group post-treatment.

### 3.3 Functional analysis of key differentially expressed proteins

An in-depth analysis and investigation into the functions of the six key differentially expressed proteins was performed. As shown in **Table 2**, subcellular localization analysis revealed that Q9NQH7 (XPNPEP3) is present not only in the nucleus but also in mitochondria. Q13243 (SRSF5) and Q13247 (SRSF6) are primarily localized to the nucleus, while P23381 (WARS1) and O75874 (IDH1) are mainly found in the cytoplasm. Q8WWA0 (ITLN1) is predominantly localized to the extracellular. In domain analysis, Q9NQH7 (XPNPEP3) contains the Aminopeptidase P, N-terminal domain (PF05195) and the Metallopeptidase family M24 (PF00557). Q13243 (SRSF5) and Q13247 (SRSF6) contain the RNA recognition motif (PF00076), P23381 (WARS1) contains the tRNA synthetases class I (PF00579) and WHEP-TRS domains (PF00458), O75874 (IDH1) contains an isocitrate/isomethylmalate dehydrogenas (PF00180), and Q8WWA0 (ITLN1) has no identified domains. None of these six proteins exhibit transcription factor functions.

**Table 2:** Functional enrichment analysis of 6 key differentially expressed proteins.

Proteins	Subcellular localization analysis	Domain analysis
Q9NQH7 (XPNPEP3)	Nucleus, Mitochondria	Aminopeptidase P, N-terminal domain (PF05195), Metallopeptidase family M24 (PF00557)
Q13243 (SRSF5)	Nucleus	RNA recognition motif (PF00076)
Q13247 (SRSF6)	Nucleus	RNA recognition motif (PF00076)
P23381 (WARS1)	Cytoplasm	tRNA synthetases class I (PF00579), WHEP-TRS domain (PF00458)
O75874 (IDH1)	Cytoplasm	Isocitrate/isopropylmalate dehydrogenase (PF00180)
Q8WWA0 (ITLN1)	Extracellular	/

### 3.4 Clinical significance of key differentially expressed proteins

**Figure 3:**
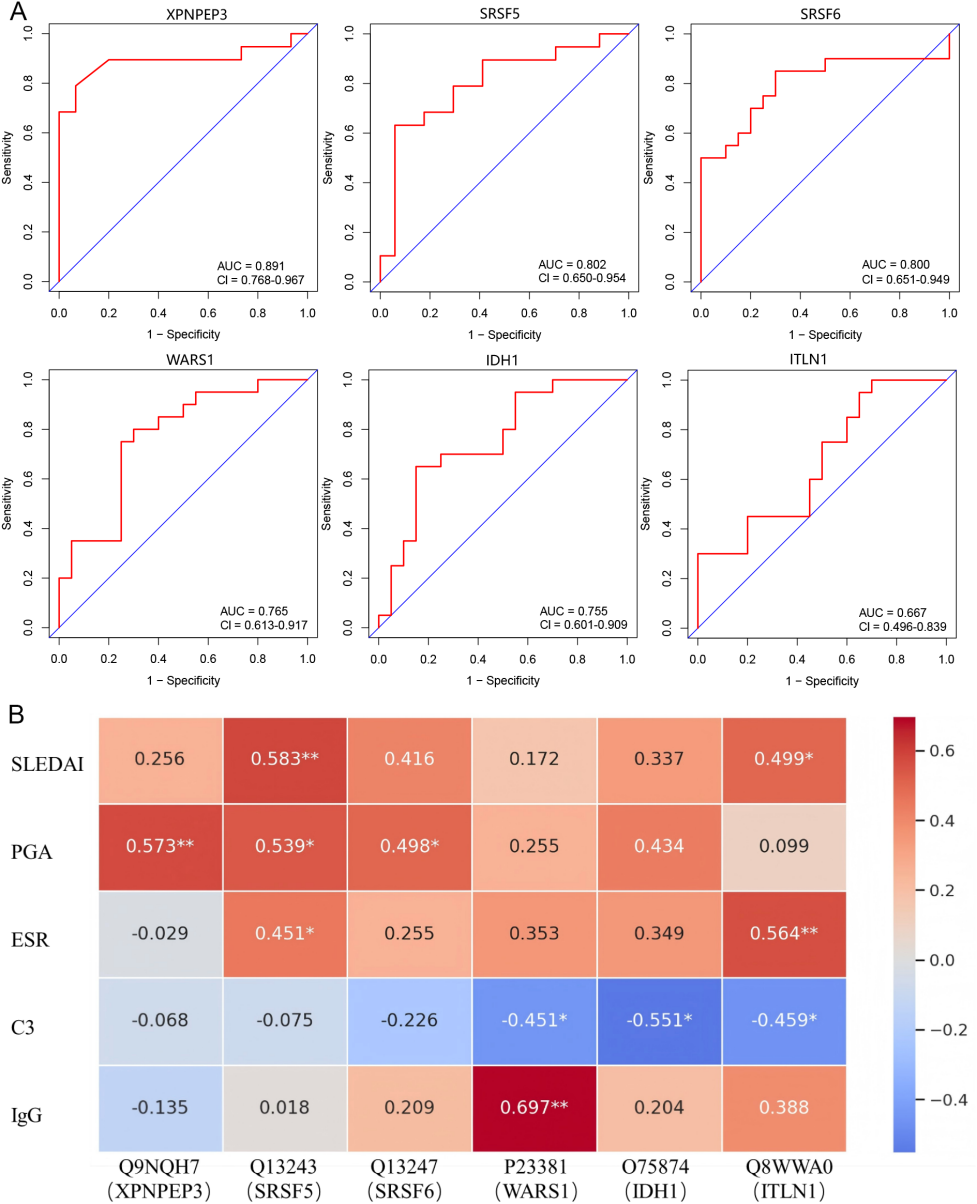
Clinical significance of key differentially expressed proteins. **(A)** The ROC curve demonstrated the predictive performance of the key differentially expressed protein Q9NQH7 (XPNPEP3), Q13243 (SRSF5), Q13247 (SRSF6), P23381 (WARS1), O75874 (IDH1), Q8WWA0 (ITLN1). **(B)** The correlation heatmap showed the relationship between key differentially expressed proteins and clinical indicators in SLE patients prior to treatment.

The ROC analysis indicated that the AUC values for the six proteins—Q9NQH7 (XPNPEP3), Q13243 (SRSF5), Q13247 (SRSF6), P23381 (WARS1), O75874 (IDH1), and Q8WWA0 (ITLN1)—were 0.891, 0.802, 0.800, 0.765, 0.755, and 0.667, respectively, with the first three proteins demonstrating higher diagnostic value (**Figure 3A**). Due to the absence of internal validation, these ROC curves represent internal, exploratory performance. We conducted a preliminary correlations analysis between these six proteins and clinical indicators associated with treatment efficacy (SLEDAI, PGA, ESR, C3, and IgG) prior to treatment in the R group (**Figure 3B**). Q9NQH7 (XPNPEP3) was positively correlated with PGA; Q13243 (SRSF5) positively correlated with SLEDAI, PGA, and ESR; Q13247 (SRSF6) positively correlated with PGA; P23381 (WARS1) showed negative correlation with complement C3 and positive correlation with IgG; O75874 (IDH1) showed negative correlation with complement C3; Q8WWA0 (ITLN1) showed positive correlation with SLEDAI and ESR, and negative correlation with complement C3. These findings indicate that these proteins may predict treatment efficacy.

### 3.5 Effect of telitacicept treatment on serum metabolites in SLE patients

With OPLS-DA VIP > 1, and P value < 0.05, significantly differentially expressed metabolites were screened (**Supplementary Table 3**). In the POS mode, 35 significantly differentially expressed metabolites were identified in the Pre-R vs Post-R group, and 4 in the Pre-NR vs Post-NR group. In the NEG mode, 17 significantly differentially expressed metabolites were identified in the Pre-R vs Post-R group, and 12 were identified in the Pre-NR vs Post-NR group.

### 3.6 Functional enrichment analysis of key differentially expressed metabolites

**Figure 4:**
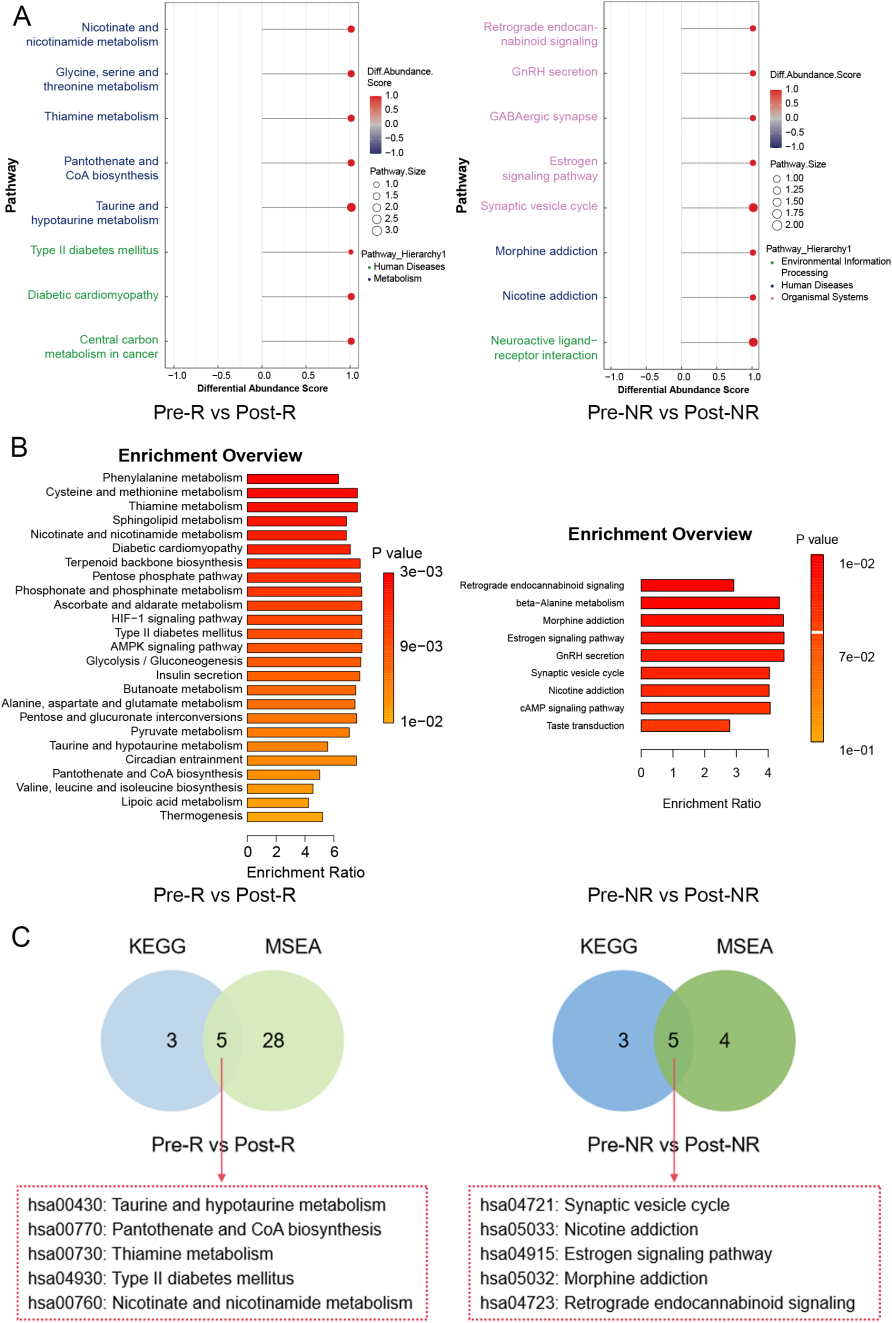
Functional enrichment analysis of metabolites. **(A)** The KEGG enrichment analysis of differentially expressed metabolites in the R and NR groups. **(B)** MSEA of metabolites in the R and NR groups. **(C)** The venn diagrams showed the common pathways between KEGG and GSEA in the R and NR groups.

Using between-group comparisons, key differentially expressed metabolites were identified between R and NR groups in both POS and NEG modes. However, no metabolites were found to be common to both modes. Therefore, we shifted our approach to jointly screen for key differentially expressed metabolites by integrating KEGG pathway analysis results with MSEA results. In the KEGG enrichment analysis, 8 pathways were significantly enriched in the Pre-R vs Post-R group, and 8 pathways were significantly enriched in the Pre-NR vs Post-NR group (P value < 0.05 & |DA Score|≥1, **Figure 4A**). The MSEA results revealed 33 pathways significantly enriched in the Pre-R vs Post-R group and 9 pathways significantly enriched in the Pre-NR vs Post-NR group (P value < 0.05 & Enrichment ratio > 2, **Figure 4B**). The venn diagrams indicated that five pathways were significantly enriched in both the Pre-R vs Post-R group and the Pre-NR vs Post-NR group (**Figure 4C**). As shown in **Table 3**, pyruvate participated in all five pathways in the Pre-R vs Post-R group, and gamma-aminobutyric acid (GABA) similarly participated in all five enriched pathways in the Pre-NR vs Post-NR group. This suggests that these two metabolites may play a crucial role in the therapeutic process of Telitacicept for SLE and could serve as key differentially expressed metabolites.

**Table 3:** Metabolites involved in pathways commonly enriched by KEGG and MSEA.

Groups	Pathways ID	Pathways name	Metabolites
Pre-R vs Post-R	hsa00430	Taurine and hypotaurine metabolism	**Pyruvate**, DL-cysteine, Guanidinoethyl sulfonate
	hsa00770	Pantothenate and CoA biosynthesis	**Pyruvate**, DL-cysteine
	hsa00730	Thiamine metabolism	**Pyruvate**, DL-cysteine
	hsa04930	Type II diabetes mellitus	**Pyruvate**
	hsa00760	Nicotinate and nicotinamide metabolism	**Pyruvate**, Nicotinate d-ribonucleotide
Pre-NR vs Post-NR	hsa04721	Synaptic vesicle cycle	**GABA**, Glycine
	hsa05033	Nicotine addiction	**GABA**
	hsa04915	Estrogen signaling pathway	**GABA**
	hsa05032	Morphine addiction	**GABA**
	hsa04723	Retrograde endocannabinoid signaling	**GABA**

GABA, Gamma-aminobutyric acid. Bolded characters highlight emphasized roles.

### 3.7 Clinical significance of key differentially expressed metabolites

**Figure 5:**
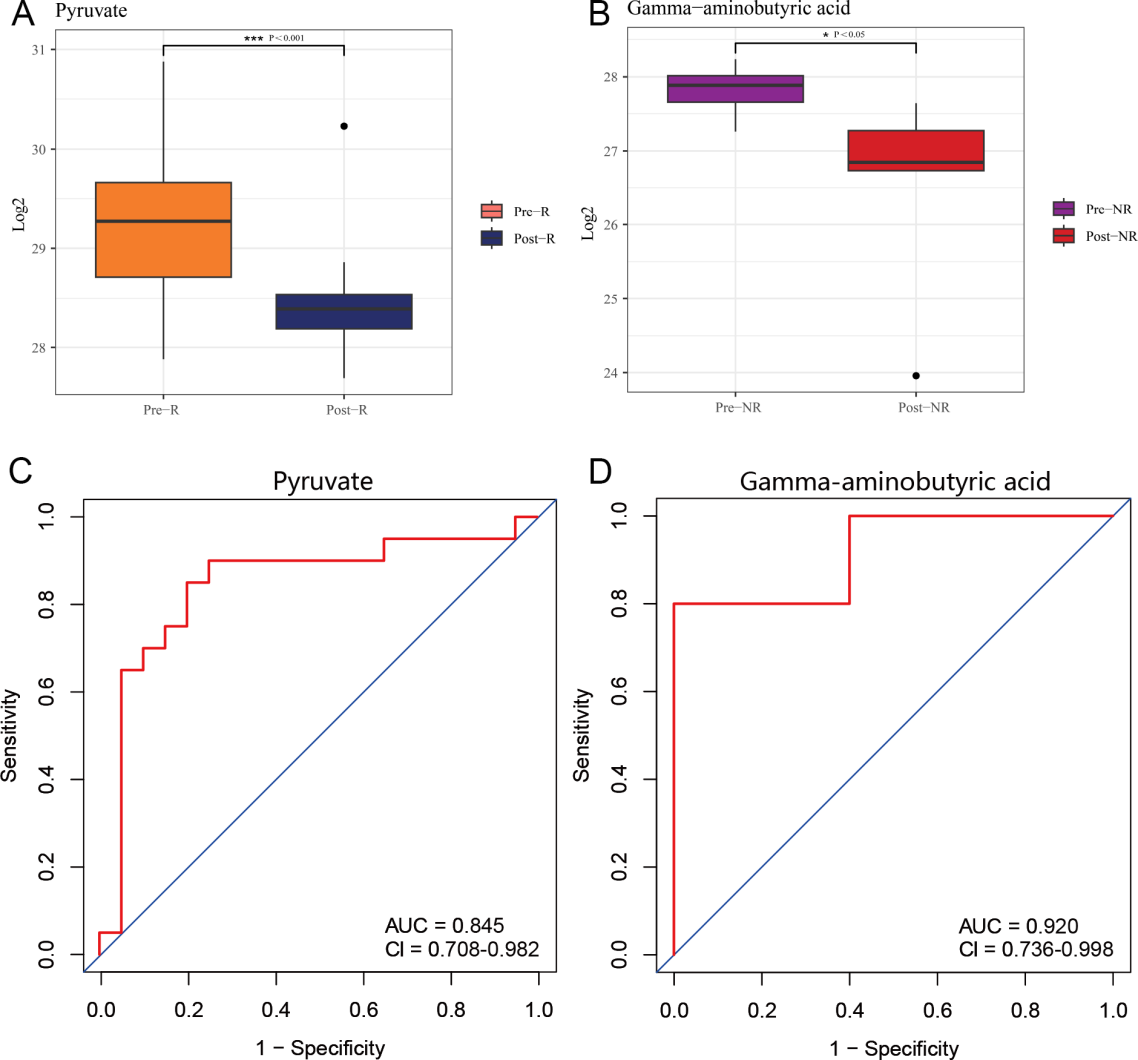
Clinical significance of key differentially expressed metabolites. **(A)** The box plot illustrated the levels of pyruvate in Pre-R vs Post-R group. **(B)** The box plot illustrated the levels of GABA in the Pre-NR and Post-NR group. **(C)** The ROC curve demonstrated the predictive performance of pyruvate. **(D)** The ROC curve demonstrated the predictive performance of GABA. * : P<0.05 *** : P<0.001.

The box plots exhibited that both pyruvate (P = 0.00039) and GABA (P = 0.01136) levels decreased post-treatment, with the change in pyruvate exhibiting high statistical significance (**Figures 5A, B**). To evaluate the value of pyruvate and GABA in clinical therapeutic efficacy, ROC analysis was conducted (**Figures 5C, D**). The AUC value of pyruvate in the Pre-R vs Post-R group was 0.845, and the AUC value of GABA in the Pre-NR vs Post-NR group was 0.920, suggesting potential diagnostic utility that required validation in independent cohorts to confirm generalizability. We conducted a preliminary analysis of the correlation between these two metabolites and pre-treatment clinical efficacy indicators (SLEDAI, PGA, ESR, C3, and IgG). The results revealed no significant correlations, with all P values exceeding 0.05. This may be attributed to sample size limitations, as the NR group contained only five samples. Second, metabolite levels are susceptible to significant variability influenced by individual differences, diet, medication, and other factors, potentially obscuring their relationship with clinical indicators. NR-associated candidate biomarkers identified through exploratory analysis require validation in larger cohorts to confirm their potential diagnostic utility, given the inherent limitations of small sample size.

### 3.8 Integrated analysis of proteomics and metabolomics

**Figure 6:**
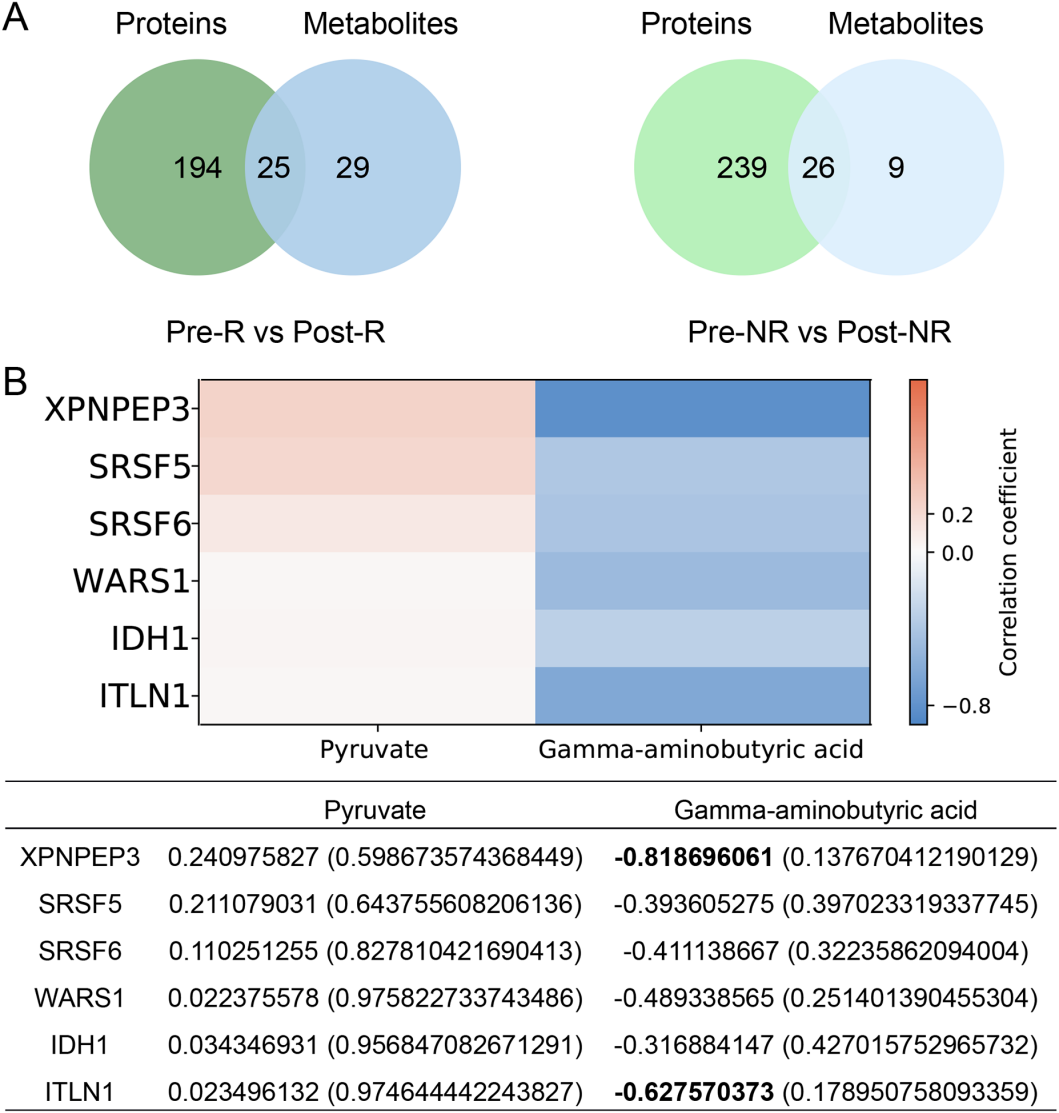
Integrated analysis of proteomics and metabolomics. **(A)** Venn plot showing the pathways shared by differentially expressed proteins and metabolites in the Pre-R vs Post-R and Pre-NR vs Post-NR groups. **(B)** Correlation analysis between 6 key differentially expressed proteins (XPNPEP3, SRSF5, SRSF6, WARS1, IDH1, and ITLN1) and 2 key differentially expressed metabolites (pyruvate and GABA). The numbers in the table indicate: r (P value).

The KEGG enrichment analysis indicated that 219 and 54 pathways were enriched by differentially expressed proteins and metabolites in the Pre-R vs Post-R group, respectively. In the Pre-NR vs Post-NR group, 265 and 35 pathways were enriched by differentially expressed proteins and metabolites, respectively. The venn diagrams revealed 25 pathways jointly involved by differentially expressed proteins and metabolites in the Pre-R vs Post-R group, and 26 pathways shared by differentially expressed proteins and metabolites in the Pre-NR vs Post-NR group (**Figure 6A**). We screened for co-occurring metabolic pathways among 6 key proteins (XPNPEP3, SRSF5, SRSF6, WARS1, IDH1, and ITLN1) and 2 key metabolites (pyruvate and GABA). In the Pre-R vs Post-R group, protein IDH1 and metabolite pyruvate were found to share two metabolic pathways: central carbon metabolism in cancer (hsa05230) and the citric acid cycle (hsa00020). In the Pre-NR vs Post-NR group, no shared metabolic pathways were identified. In addition, the correlation analysis between 6 key proteins and 2 key metabolites were performed, indicating that all six proteins exhibited positive correlations with pyruvate and negative correlations with GABA (**Figure 6B**). Among these, XPNPEP3 and GABA (r = −0.82) and ITLN1 and GABA (r = −0.63) demonstrated relatively high negative correlation coefficients. However, their statistical significance was not directly confirmed in this study and requires further validation with increased sample size.

## 4 Discussion

Aberrant activation and differentiation of B cells in SLE drive the generation of multiple autoantibodies and immune complex deposition, thereby triggering tissue inflammation and damage. This study demonstrates that Telitacicept, a dual B-cell targeting agent acting on BAFF and APRIL, improves laboratory parameters in the majority of SLE patients. The observed improvements in the R group directly support the efficacy of Telitacicept, whereas the NR group failed to achieve expected therapeutic outcomes, indicating that Telitacicept is not universally effective across all SLE patients in real-world settings, necessitating further investigation into the underlying mechanisms of variable response. Consequently, while Telitacicept represents a novel therapeutic option for SLE, its variable efficacy underscores the imperative to develop more precise B lymphocyte-targeting strategies to achieve personalized treatment.

Proteomics profiling identified six protein biomarkers, including XPNPEP3, SRSF5, SRSF6, WARS1, IDH1, and ITLN1, with potential predictive value for therapeutic response in SLE patients. These proteins exhibited high expression pre-treatment in the R group and low expression post-treatment, whereas the NR group showed inverse expression profiles. The observed upregulation in the NR group may to a greater extent reflect that the treatment failed to reverse the baseline-related proteomic characteristics. XPNPEP3 is a transcriptional target of canonical Wnt/β-catenin signaling (26). XPNPEP3 may be involved in the immune regulation and inflammatory response of SLE through the Wnt/β-catenin signaling pathway. Although there is no direct evidence for the role of splicing factor SRSF5 in SLE, it may influence disease onset by regulating the alternative splicing of immune-related genes (27). SRSF6 and SRSF5 are both splicing factors that have been implicated in other autoimmune diseases (osteoarthritis and systemic sclerosis), associated with inflammation or susceptibility (28, 29). It is hypothesized that they may also contribute to SLE pathogenesis by regulating the splicing of inflammatory factors and abnormal B-cell activation. WARS1 enhances the immunomodulatory capacity of mesenchymal stem cells by maintaining the RhoA-Akt signaling axis (30). Its high expression is closely associated with immune cell activation, consistent with the abnormal immune activation state observed in SLE patients. Metabolites generated by IDH1 mutations may influence the epigenetic state of immune cells, thereby affecting their response to inflammatory signals (31). ITLN1 enhances anti-inflammatory capacity and promotes M2 macrophage polarization in rheumatoid arthritis by stimulating synovial fibroblasts to increase interleukin-4 expression (32). M1 macrophages are involved in the progression of active lupus nephritis (LN), while M2 macrophages are expressed during the remission phase of LN (33). Thus, we inferred that ITLN1 may regulate inflammation by promoting M2 macrophage polarization, suggesting its potential value in determining SLE disease outcomes and patient stratification. These exploratory findings identify candidate proteins correlated with treatment efficacy that warrant further validation in independent cohorts to confirm their diagnostic potential and clinical utility.

Metabolomics profiling indicates that pyruvate is a potential metabolic biomarker for the R group to Telitacicept in treating SLE, while GABA is a potential metabolic biomarker for the NR group. Pyruvate primarily participates in energy metabolism processes. A metabolomics study revealed that pyruvate was increased in SLE mouse brains, which was further increased after prednisone treatment (34). It contrasts with our findings, as pyruvate decreased in SLE patients after treatment with Telitacicept. This discrepancy may stem from the differing mechanisms of action between the two drugs, as well as variations in study subjects—this research focused on SLE patients, whereas the literature utilized mouse models. We speculate that the decreased pyruvate may potentially influence B-cell activation and metabolism via the citric acid cycle, thereby improving disease status in SLE patients. Consequently, pyruvate may serve as a monitoring indicator for Telitacicept efficacy in SLE. GABA is an inhibitory neurotransmitter. Genetic polymorphisms in the GABA receptor-associated protein had been linked to SLE susceptibility (35). Some SLE patients may also develop neurological complications, known as neuropsychiatric lupus. Researches indicate that anti-GABA receptor antibodies may serve as potential biomarkers for assessing disease activity and prognosis in such patients (36, 37). In this study, the level of GABA decreased after treatment in the NR group. We boldly hypothesize that this phenomenon may indicate weakened inhibitory effects of GABA in the NR group, suggesting impaired neurological function and inadequate disease control. Due to the limited sample size, especially in the NR group, these findings need to be interpreted with caution and further in-depth research is necessary.

**Figure 7:**
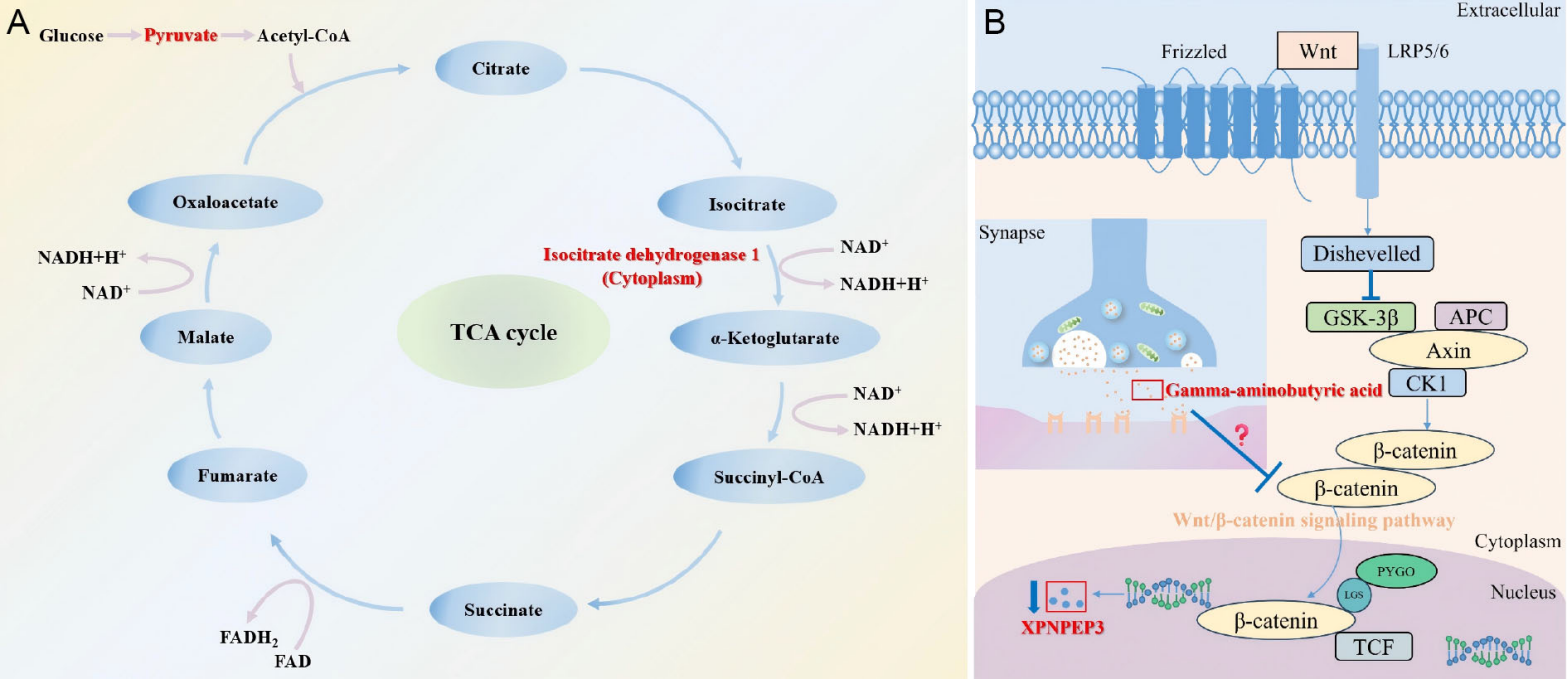
Reasonable speculation of joint analysis. **(A)** The citric acid cycle involving IDH1 and pyruvate. **(B)** Hypothetical diagram of gamma-aminobutyric acid negatively regulating XPNPEP3 through the Wnt/β-catenin signaling pathway. This schematic illustrates a hypothesis-generating pathway based on correlational and literature-derived evidence. Direct functional validation is required to confirm causality.

In this analysis, KEGG pathway analysis indicated that protein IDH1 and metabolite pyruvate participated in the metabolic pathways of central carbon metabolism in cancer (hsa05230) and the citric acid cycle (hsa00020) in the R group. Central carbon metabolism serves as the core pathway for cellular energy metabolism and biosynthesis, primarily encompassing glycolysis, the citric acid cycle, the pentose phosphate pathway, glutamine metabolism, and lipid metabolism (38). As shown in **Figure 7A**, the protein IDH1 and the metabolite pyruvate participate in the citric acid cycle. IDH1 plays a crucial role in regulating cellular metabolism and redox status. Its metabolite, α-ketoglutarate, participates in the citric acid cycle, influencing epigenetic regulation and thereby affecting the function of immune cells (39, 40). Furthermore, reduced entry of pyruvate into the citric acid cycle impairs the production of type I interferon by plasmacytoid dendritic cells in SLE patients (41). B cells can also influence humoral immunity by regulating the balance between substrates, enzymes, and energy requirements in the citric acid cycle (42). Therefore, Telitacicept may regulate pyruvate levels to affect B-cell activation and metabolism through the citric acid cycle, ultimately improving the condition of SLE patients. The IDH1/pyruvate axis showed directionally consistent dynamic changes at both the protein and metabolite levels, and was highly related to known biological processes, enabling it a representative example for mechanism explanation. The correlation analysis showed a negative correlation between XPNPEP3 and GABA in NR group, which may suggest a potential regulatory relationship. XPNPEP3 is a potential regulatory target of the Wnt/β-catenin signaling pathway (26). Activation of the Wnt signaling pathway promotes the transcription of XPNPEP3. We speculate that GABA, as an inhibitory neurotransmitter, may indirectly reduce XPNPEP3 production by inhibiting β-catenin activity (**Figure 7B**). The role of IDH1 in the citric acid cycle and its metabolic/epigenetic regulatory functions are mechanistically validated through decades of biochemical and genetic studies. In contrast, the GABA–Wnt/β-catenin–XPNPEP3 axis remains a hypothesis requiring functional validation. Future studies should employ functional genomics and pharmacologic interventions to validate these mechanistic hypotheses.

The limitations of this study must be considered to contextualize the findings appropriately. First, the small sample size, particularly in the NR group (n = 5) compared to the R group (n = 20)—a 4:1 imbalance—severely limits statistical power and the reliability of results. Second, the absence of internal validation (e.g., cross-validation or split-sample testing) risks overestimating ROC curve performance and predictive accuracy of the identified biomarkers, potentially inflating their clinical relevance. Third, all patients received Telitacicept combined with standard treatment, introducing confounding effects that may obscure or magnify true differences in protein/metabolite profiles between R and NR groups, complicating causal interpretation. Observed changes in pyruvate and GABA levels may reflect combined effects of Telitacicept and concomitant therapies, necessitating future studies with stratified cohorts to disentangle these contributions. Fourth, the use of P <0.05 as the threshold for differential expression may elevate false-positive risk. Fifth, the lack of baseline clinical and molecular comparisons between the R and NR groups precludes distinguishing pre-existing, treatment-independent differences from treatment-induced changes. Finally, while the six proteins (XPNPEP3, SRSF5, SRSF6, WARS1, IDH1, ITLN1) show mechanistic promise, their translation to clinical application remains distant, requiring validation in larger, independent cohorts with rigorous statistical modeling. These limitations underscore the need for cautious interpretation of the exploratory findings and emphasize the importance of replication in well-powered studies to confirm their utility in precision medicine for SLE.

## 5 Conclusion

In summary, this study employs a multi-omics integrated analysis to thoroughly investigate the therapeutic biomarkers and differential mechanisms of Telitacicept in treating SLE. The differential expression patterns of proteins and metabolites provide mechanistic insights into therapeutic response variability, paving the way for precision medicine approaches in SLE management. Moving forward, larger-scale clinical validation and deeper mechanistic research hold promise for advancing precision medicine in SLE and provide broader scientific evidence for targeted therapies in autoimmune diseases.

## Data Availability

The mass spectrometry proteomics data have been deposited to the ProteomeXchange Consortium (http://proteomecentral.proteomexchange.org) via the iProX partner repository with the dataset identifier PXD073923 (https://proteomecentral.proteomexchange.org/cgi/GetDataset?ID=PXD073923). The raw metabolomics data are accessible on the OMIX platform under the dataset ID OMIX014815-01 (https://ngdc.cncb.ac.cn/omix/preview/EZckq3x1).
