# Supplementary material for: Unveiling biomarkers of telitacicept’s efficacy in SLE treatment through proteomics and metabolomics
Source: Front Immunol. 2026 Mar 4;17:1779880. doi: 10.3389/fimmu.2026.1779880 (PMC12996235; doi:10.3389/fimmu.2026.1779880)
Supplement: Supplementary file 1 [file Table1.docx]

Supplementary Material

**Supplementary Table 1**

| **SLE Patient Inclusion and Exclusion Criteria** | |
| --- | --- |
| **SLE Patient Inclusion Criteria** | |
| (1) | Aged between 18 and 75 |
| (2) | Patients diagnosed with active SLE must meet at least 4 of the 11 criteria in the 1997 revised SLE classification criteria established by the American College of Rheumatology (ACR) |
| (3) | Patient has received standard treatment for SLE for at least 3 months, and the types and doses of treatment medications have remained stable for at least 30 days |
| (4) | Patients with SLE who have demonstrated good compliance in the past, fully understand the relevant information about the treatment regimen, agree to the regular use of Telitacicept, and sign the informed consent form |
| (5) | Positive ANA antibodies and a SLEDAI score ≥ 8 (or a SLEDAI score ≥ 6 if low complement levels and/or positive anti-dsDNA antibodies are present) |
| **SLE Patient Exclusion Criteria** | |
| (1) | Severe nephritis (proteinuria >6 g/24h or serum creatinine >221 μmol/L within the past 2 months) or requiring hemodialysis |
| (2) | Severe central nervous system disease caused by SLE or non-SLE within the past 2 months |
| (3) | Severe diseases of the circulatory system, digestive system, blood system, and endocrine system |
| (4) | Prednisone ≥100 mg/day for more than 14 days or plasma exchange within one month |
| (5) | Participation in any other biologic therapy within the past 12 months |
| (6) | Pregnant or breastfeeding women |

SLE: systemic lupus erythematosus; SLEDAI: Systemic Lupus Erythematosus Disease Activity Index.
